# Undifferentiated Pleomorphic Sarcoma with Reactive Eccrine Syringofibroadenoma: A Case Report

**DOI:** 10.3390/dermatopathology11040030

**Published:** 2024-10-20

**Authors:** Navinda Donsakul, Suthep Jerasutus, Ittipon Tubtieng, Ravion Assavanatenapa, Voraphol Vejjabhinanta

**Affiliations:** 1 Southern Regional Hospital of Tropical Dermatology-Trang Province, Department of Medical Services, Ministry of Public Health, Trang 92000, Thailand; 2 Division of Dermatology, Department of Medicine, Faculty of Medicine Ramathibodi Hospital, Mahidol University, Bangkok 10400, Thailand; 3 Phatthalung Hospital, Phatthalung 93000, Thailand; 4 Institute of Dermatology, Department of Medical Services, Ministry of Public Health, Bangkok 10400, Thailand

**Keywords:** undifferentiated pleomorphic sarcoma, eccrine syringofibroadenoma, malignant fibrous histiocytoma, soft tissue sarcoma

## Abstract

Undifferentiated pleomorphic sarcoma (UPS) is an aggressive soft tissue sarcoma with a poor prognosis. The patients are usually found to have metastasis when the primary tumor is diagnosed. Eccrine syringofibroadenoma (ESFA) is a rare cutaneous adnexal lesion of eccrine duct origin. There are five subtypes, one of which is reactive ESFA, known to occur in reaction to an inflammatory or neoplastic process. In this article, we report a case of the co-existence of both UPS and ESFA in a 70-year-old male patient, presenting with a painless, erythematous, irregular surface nodule with a peripherally extended brownish hyperkeratotic plaque on the right palm. The histologic findings revealed an ill-defined dermal tumor of atypical epithelioid and spindle-shaped cells with large pleomorphic hyperchromatic nuclei and abundant eosinophilic cytoplasm. Some of those cells were multinucleated giant cells in the stroma with vascular proliferation and mixed inflammatory cell infiltrate. The tumor cells, which were only positive for vimentin, supported the diagnosis of undifferentiated pleomorphic sarcoma (UPS). Meanwhile, the overlying epidermis demonstrated hyperkeratosis, papillated epidermal hyperplasia, and proliferation of anastomosing slender cords and strands of cuboid cells within loose fibrovascular stroma. These findings are the characteristics of eccrine syringofibroadenoma (ESFA). We describe here a patient in whom reactive ESFA occurred on and surrounded the UPS tumor.

## 1. Introduction

Undifferentiated pleomorphic sarcoma (UPS), previously termed malignant fibrous histiocytoma, is a neoplasm of soft tissues. The patients usually present with a painless, abruptly growing circumscribed mass on extremities. It is reported to affect males more than females. The most common age group is 50 to 70 years old. It often involves the lower and upper extremities, while other parts, such as the chest wall, retroperitoneum, abdomen, head, and neck, can also be found [1,2].

Eccrine syringofibroadenoma (ESFA) is a rare cutaneous adnexal lesion of eccrine duct origin. However, it remains unclear whether ESFA is a neoplastic process, hamartoma, or reactive eccrine hyperplasia [3]. In this report, we present a rare case of a patient with both UPS and reactive ESFA on the palm. To the best of our knowledge, the co-existence of UPS and reactive ESFA has never been reported in English literature so far.

## 2. Case Report

**Figure 1:**
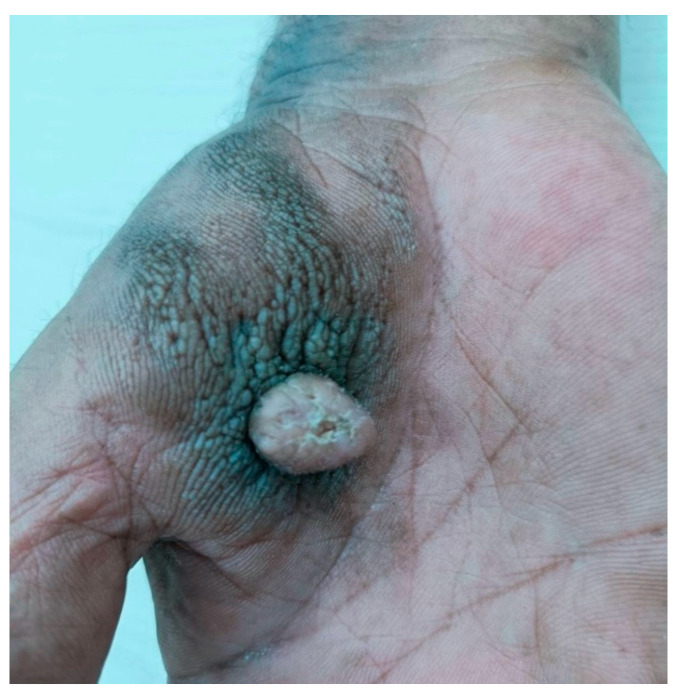
A firm, faint erythematous verrucous nodule size of 1 × 1 cm, surrounded by a brownish infiltrative plaque on the right palm.

A 70-year-old male patient with no known underlying disease noticed a painless erythematous irregular surface nodule on his right palm for five years. The nodule enlarged more rapidly, developing a peripherally extended brownish hyperkeratotic plaque within one year. Dermatological examination revealed a firm, faint erythematous verrucous nodule size of 1 × 1 cm, surrounded by a brownish infiltrative plaque on the right palm (Figure 1).

**Figure 2:**
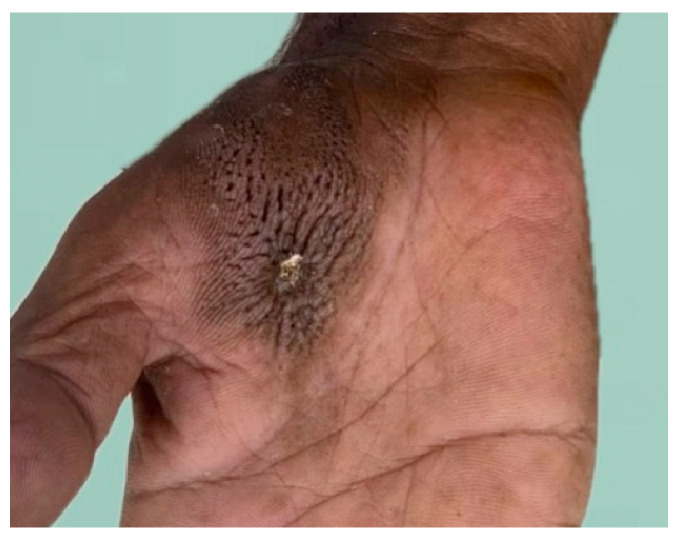
Two weeks after the excision of the nodule with some part of the brownish hyperkeratotic plaque on the right palm.

An excisional biopsy was performed, and then the entire nodule, with some part of the brownish hyperkeratotic plaque located inferior to the nodule, was sent for histopathological evaluation. The remaining lesion were shown in Figure 2.

**Figure 3:**
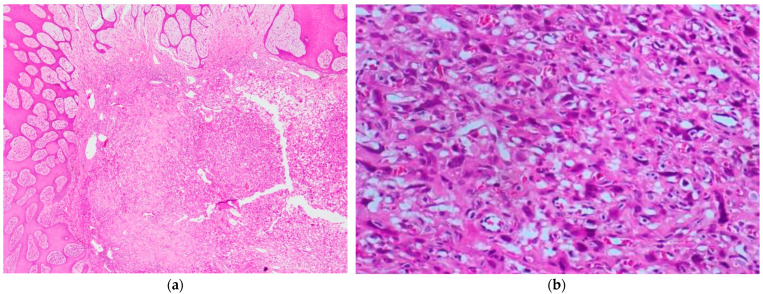
(**a**) Scanning view demonstrating an ill-defined, densely cellular dermal tumor affecting the entire dermis without any connection to the overlying epidermis (Hematoxylin and Eosin, ×20). (**b**) Medium-power view demonstrating the tumor composed of atypical epithelioid and spindle-shaped cells with marked pleomorphic hyperchromatic or vesicular nuclei (Hematoxylin and Eosin, ×100).

**Figure 4:**
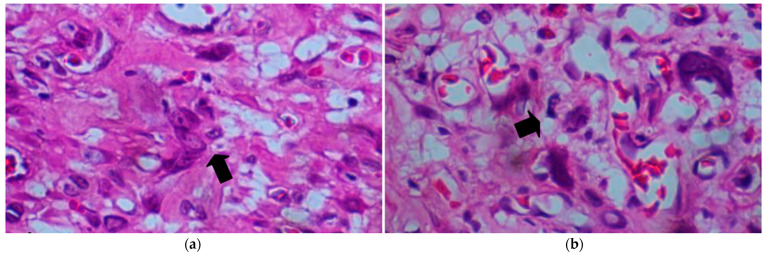
(**a**) Tumor cells show pleomorphic nuclei and abundant cytoplasm as well as multinucleated giant cells (black arrow). (**b**) Tumor cells exhibit marked pleomorphic bizarre-shaped nuclei; some atypical mitotic figures are occasionally observed (black arrow) (Hematoxylin and Eosin, ×400).

Microscopic examination revealed an ill-defined, densely packed cellular tumor occupying the entire dermis and invading beyond the base of the biopsy specimen without any connection to the overlying epidermis. The tumor consisted of pleomorphic epithelioid and spindle-shaped cells. The tumor cell morphologies were markedly atypical, with large hyperkeratotic irregular or vesicular nuclei and prominent nucleoli, multiple mitoses, including atypical mitotic figures, and multinucleated giant cells (Figure 3 and Figure 4). The stroma consisted of delicate vasculature, focal myxoid changes, and a mixture of acute and chronic inflammatory cells.

**Figure 5:**
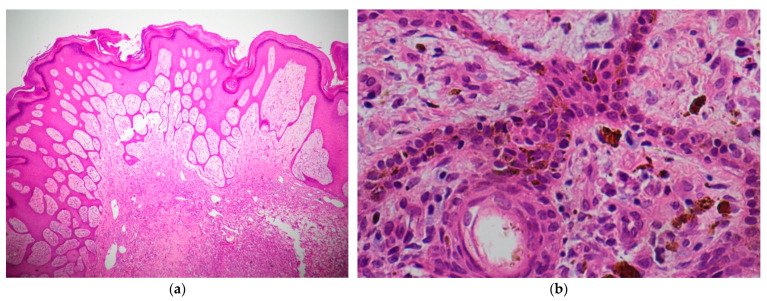
(**a**) Scanning view demonstrating hyperkeratosis, papillomatosis, and proliferation of thin anastomosing slender cords and strands of epithelial cells from multiple foci of the epidermis, enclosing loose fibrovascular stroma (Hematoxylin and Eosin, ×20). (**b**) High-power view demonstrating the epithelial strands, which consist of uniform poroid cells with ductal differentiation (Hematoxylin and Eosin, ×200).

The overlying epidermis showed hyperkeratosis, papillated epidermal hyperplasia, and a proliferation of anastomosing slender epithelial cords and strands from multiple foci of the epidermis, enclosed in loose fibrovascular stroma. The epithelial strand consisted of poroid cells, some of which showed ductal lumina rimmed by a homogeneous eosinophilic cuticle (Figure 5).

An immunohistochemical analysis was performed, revealing that the tumor cells were strongly positive only for vimentin. The other markers, including cytokeratin AE1/AE3, EMA, S100 protein, SMA, desmin, CD10, CD34, and CD68, were all negative.

On the basis of the histological and immunohistochemical analysis, the diagnosis of undifferentiated pleomorphic sarcoma with reactive eccrine syringofibroadenoma was made. After the excisional biopsy, the patient underwent magnetic resonance imaging (MRI) of the right hand for further investigation. The study revealed mild edema of the overlying subcutaneous fat tissue at the excised area without a detectable mass. There was an enhancing nodule size of 0.7 × 0.3 × 0.2 cm at the ulnar dorsal side of the first proximal phalangeal shaft. The lesion abutted and caused cortical erosion at the proximal phalanx. The mass was located deep in the extensor pollicis longus tendon. Further tissue diagnosis or monitoring mass progression with MRI was recommended to rule out metastasis. The computed tomography scan of the chest and upper abdomen revealed no detectable pulmonary nodule, significant lymphadenopathy or bone metastasis. However, low-density liver nodule sizes of 0.8 × 0.3 cm at segments 4 to 8 and 0.5 × 0.4 cm at segment 5 were found, which required further investigation for metastasis and staging. A liver biopsy and wider excision of the tumor on the palm were initially planned. Unfortunately, the patient was lost to follow-up.

## 3. Discussion

UPS is one of the most common subtypes of soft tissue sarcoma, previously known as malignant fibrous histiocytoma (MFH) [2].

MFH, previously known as a soft tissue sarcoma, was considered to be of fibrohistiocytic lineage. The term MFH is no longer in use after careful pathologic and immunohistochemical analysis showed no evidence of true histiocytic differentiation. It was replaced by the term ‘UPS’ [4]. Many lesions previously called MFH have been reclassified into lineage-specific sarcomas such as liposarcoma, leiomyosarcoma, rhabdomyosarcoma, malignant peripheral nerve sheath tumor, and osteosarcoma [5,6]. Currently, the World Health Organization has classified the existence of UPS in the category of soft tissue tumors of uncertain differentiation [7].

UPS represents about 5% of all soft tissue sarcomas. The tumors commonly occur on the lower and upper extremities as deep-seated tumors that usually affect adults aged 50–70 years old [1,8]. UPS includes not only neoplasm from the skin but also sarcoma from internal organs, retroperitoneal, and osteoid origins [1].

Upon reviewing our patient’s detailed history, it was noted that five years ago, the patient observed a small lump on his palm. It was initially believed to be a wart and was treated topically, which resulted in some improvement, although it was never fully resolved.

Approximately one year ago, the patient noticed the lump had grown, and there was also a surrounding brown discoloration. At this point, we cannot conclusively determine whether the events from five years ago are related to the current condition. The exact duration of the tumor’s presence remains unclear, and thus we cannot definitively assess whether there has been a lack of rapid growth. Typically, UPS presents as a painless, enlarging mass, often over a period of several months, although in some cases, the duration may be significantly longer depending on the rate of growth. A rapid increase in growth usually prompts patients to seek medical attention promptly.

The histopathological appearance of the tumor is not very consistent, other than showing closely packed tumor cells with various morphologies of atypical spindle-shaped cells and epithelioid cells with pleomorphic, hyperchromatic, or vesicular nuclei, as well as atypical multinucleated giant cells and often atypical mitotic figures.

Differential diagnoses may include dermatofibroma (DF) with monster cells, atypical fibroxanthoma (AFX), poorly differentiated squamous cell carcinoma, melanoma, and other sarcomas that commonly affect the skin.

UPS may be confused with DF with monster cells, a subtype of early-evolving lesion. Although DF with monster cells may display strikingly atypical mononuclear cells or multinucleated giant cells [9,10], it is distinguished from UPS by its typical histologic features of DF in the background, such as epidermal hyperplasia with broad rete ridge elongation, epidermal hyperpigmented fibrohistiocyte proliferation, including sederophages and lipophages, which interpose among keloidal collagen bundles, and perivascular lymphocytic infiltrate at the periphery of the lesion [11].

Atypical AFX histologically resembles UPS, which consists of atypical spindle-shaped and epithelioid tumor cells with marked pleomorphic nuclei [12,13,14]. However, AFX is usually relatively well-demarcated and confined to the dermis [1]; furthermore, it has negative reactivity for CD10 and CD68 [14]. In our patient, the negative CD10 and CD68 help exclude AFX.

The negative reactivity of tumor cells for other lineage-specific markers, such as cytokeratin AE1/AE3, S100 protein, CD34, and SMA/desmin, allows distinction from poorly differentiated squamous cell carcinoma, melanoma, angiosarcoma, and leiomyosarcoma, respectively.

In this patient, the lesion appears to be a small, superficial 1 × 1 cm mass due to the visible exophytic portion. However, the tumor has invaded beyond the subcutaneous tissue, reaching the bone and tendon, which is inconsistent with the typical presentation of AFX, where the lesion is usually confined to the dermis. Moreover, the lower portion of the lesion extends widely beyond the superficial nodule, beneath a large overlying eccrine tumor (ESFA), measuring 4 × 5 cm.

Several factors, such as tumor depth, size, histological subtype, the patient’s age, and local recurrence, consistently correlate with prognosis, including metastasis and survival. For instance, tumors that are low-grade (<5 cm deep) with a histologic myxoid subtype and occur in patients under 50 years of age are associated with a more favorable prognosis. However, in this case, prognosis cannot be predicted solely based on histopathology. Long-term follow-up is necessary, as local recurrence and distant metastases typically develop within 12–24 months, although only a minority of patients develop metastases after five years [15].

ESFA is a rare, benign, cutaneous adnexal lesion of the acrosyringium of eccrine sweat ducts. It has variable clinical appearances, ranging from a solitary papule or nodule to multiple lesions with palmoplantar distribution or keratoderma [16]. ESFA is categorized into 5 subtypes according to its clinical presentation: (1) solitary ESFA, (2) multiple ESFA associated with anhidrotic ectodermal dysplasia, (3) multiple ESFA without associated cutaneous findings, (4) non-familial unilateral linear ESFA, and (5) reactive ESFA associated with an inflammatory or neoplastic process [17,18].

Reactive ESFA is probably a consequence of repeated damage to the eccrine sweat ducts initiated from the primary process and thus contributing to cellular proliferation.

It can occur in association with chronic inflammation such as skin ulcers, chronic lymphedema, venous stasis, burn scars, lichen planus, leprosy, and bullous pemphigoid [19,20,21,22,23]. It can be rarely associated with preexisting malignant skin tumors such as squamous cell carcinoma, Bowen’s disease, basal cell carcinoma, porocarcinoma, and Merkel cell carcinoma [24,25,26,27,28]. Up to date, the associated reactive ESFA with UPS has not been reported. This article presents a case of reactive ESFA that occurred in association with UPS.

## Data Availability

The original contributions presented in the study are included in the article, further inquiries can be directed to the corresponding authors.
